# Impact of bullying & cyberbullying on body image and disordered eating in young adult females

**DOI:** 10.1186/2050-2974-3-S1-P18

**Published:** 2015-11-23

**Authors:** Ross King, Rebecca Moorfoot, Mary Kotronakis

**Affiliations:** 1 School of Psychology, Deakin University, Victoria, Australia

## 

Weight related teasing, a verbal form of bullying, is an established predictor of body dissatisfaction and disordered eating. However, as many as 11% of university students report experiencing other forms of bullying, such as physical, social, and cyberbullying which has been linked to depression, anxiety and substance abuse. The current study aims to establish the impact of other forms of bullying on body dissatisfaction and disordered eating. Female participants (123) aged 18 – 25 years completed an anonymous online survey comprising measures of the frequency and effect of teasing, other forms of bullying, negative affect, social comparison, internalisation, body dissatisfaction, and disordered eating. Significant correlations existed between weight-related teasing, physical bullying frequency and social bullying effect but not cyberbullying and body dissatisfaction, drive for thinness and bulimic symptoms. Internalisation, appearance-based social comparison, and levels of negative affect were examined as mediators of the relationships between social and physical bullying with bulimia, drive for thinness and body dissatisfaction with support for partial or full mediation identified for many relationships. These findings further our understanding of the broader consequences of bullying and the factors that mediate this relationship, and can assist in the development of targeted preventative strategies to minimise these impacts.

